# Diethyl 2,6,11-trioxo-2,3-dihydro-1*H*-anthra[1,2-*d*]imidazole-1,3-diacetate

**DOI:** 10.1107/S1600536811028091

**Published:** 2011-07-23

**Authors:** Zahra Afrakssou, Amal Haoudi, Frédéric Capet, Christian Rolando, Lahcen El Ammari

**Affiliations:** a Laboratoire de Chimie Organique Appliquée, Université Sidi Mohamed Ben Abdallah, Faculté des Sciences et Techniques, Route d’Immouzzer, BP 2202 Fès, Morocco; b Unité de Catalyse et de Chimie du Solide (UCCS), UMR 8181, Ecole Nationale Supérieure de Chimie de Lille, France; c USR 3290 Miniaturisation pour l’Analyse, la Synthèse et la Protéomique, 59655 Villeneuve d’Ascq Cedex, Université Lille 1, France; d Laboratoire de Chimie du Solide Appliquée, Faculté des Sciences, Université Mohammed V-Agdal, Avenue Ibn Battouta, BP 1014, Rabat, Morocco

## Abstract

The title compound, C_23_H_20_N_2_O_7_, consists of three fused six-membered rings (*A*, *B* and *C*) and one five-membered ring (*D*), linked to two ethyl acetate groups. The four fused rings are slightly folded around the O=C⋯C=O direction of the anthraquinone system, with a dihedral angle of 3.07 (8)° between the fused five- and six-membered rings (*C* and *D*) and the terminal ring (*A*). The planes through the atoms forming each acetate group are nearly perpendicular to the mean plane of the anthra[1,2-*d*]imidazole system, as indicated by the dihedral angles between them of 79.94 (9) and 85.90 (9)°. The crystal packing displays non-classical C—H⋯O hydrogen bonds.

## Related literature

For the biological activity of anthraquinone derivatives, see: Afrakssou *et al.* (2011 ▶); Guimarães *et al.* (2009 ▶); Zoń *et al.* (2003 ▶). For their applications as colourants, see: Mori *et al.* (1990 ▶); Kowalczyk *et al.* (2010 ▶); Ossowski *et al.* (2005 ▶).
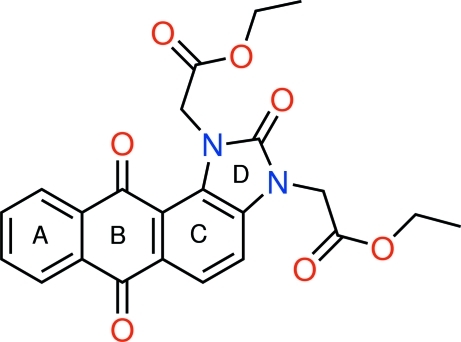


## Experimental

### 

#### Crystal data

C_23_H_20_N_2_O_7_
*M* *_r_* = 436.41
Monoclinic, 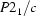

*a* = 17.462 (1) Å
*b* = 13.0646 (9) Å
*c* = 9.1411 (6) Å
β = 103.915 (3)°
*V* = 2024.2 (2) Å^3^
*Z* = 4
Mo *K*α radiation
μ = 0.11 mm^−1^
*T* = 100 K
0.49 × 0.11 × 0.09 mm

#### Data collection

Bruker APEXII CCD diffractometer
Absorption correction: multi-scan (*SADABS*; Sheldrick, 2008*a* ▶) *T*_min_ = 0.602, *T*_max_ = 0.746
32055 measured reflections
5006 independent reflections
3339 reflections with *I* > 2σ(*I*)
*R*_int_ = 0.057

#### Refinement

*R*[*F* ^2^ > 2σ(*F* ^2^)] = 0.054
*wR*(*F* ^2^) = 0.153
*S* = 1.01
5006 reflections
291 parameters
H-atom parameters constrained
Δρ_max_ = 0.57 e Å^−3^
Δρ_min_ = −0.32 e Å^−3^

### 

Data collection: *APEX2* (Bruker, 2009 ▶); cell refinement: *SAINT-Plus* (Bruker, 2009 ▶); data reduction: *SAINT-Plus*; program(s) used to solve structure: *SHELXS97* (Sheldrick, 2008*b* ▶); program(s) used to refine structure: *SHELXL97* (Sheldrick, 2008*b* ▶); molecular graphics: *ORTEP-3 for Windows* (Farrugia, 1997 ▶) and *ORTEPIII* (Burnett & Johnson, 1996 ▶); software used to prepare material for publication: *WinGX* (Farrugia, 1999 ▶).

## Supplementary Material

Crystal structure: contains datablock(s) I, global. DOI: 10.1107/S1600536811028091/bt5578sup1.cif

Structure factors: contains datablock(s) I. DOI: 10.1107/S1600536811028091/bt5578Isup2.hkl

Supplementary material file. DOI: 10.1107/S1600536811028091/bt5578Isup3.cml

Additional supplementary materials:  crystallographic information; 3D view; checkCIF report

## Figures and Tables

**Table 1:** Hydrogen-bond geometry (Å, °)

*D*—H⋯*A*	*D*—H	H⋯*A*	*D*⋯*A*	*D*—H⋯*A*
C5—H5⋯O1^i^	0.95	2.49	3.353 (2)	151
C12—H12⋯O6^ii^	0.95	2.56	3.380 (2)	145
C16—H16*A*⋯O6^ii^	0.99	2.47	3.369 (3)	151
C16—H16*B*⋯O4^ii^	0.99	2.22	3.120 (2)	151

Symmetry codes: (i) 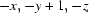; (ii) 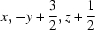.

**Fig. 1.**
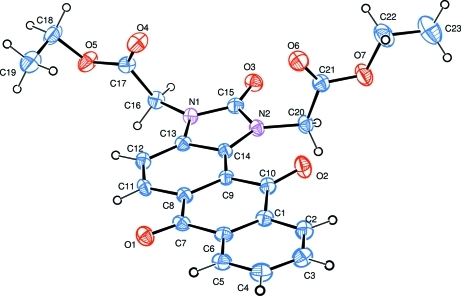
Molecular plot of the title compound with the atom-labelling scheme. Displacement ellipsoids are drawn at the 50% probability level. H atoms are represented as small circles.

## supplementary crystallographic information

### Comment

Anthraquinones, the largest group of naturally occurring quinones, present in bacteria, fungi and many higher plant families contain π-electrons, reducible *p*-quinone system and are redoxactive (Zoń *et al.*, 2003). That is the reason why they found many practical applications (Kowalczyk *et al.*, 2010; Ossowski *et al.*, 2005). Both natural and synthetic derivatives have been used as colourants in food, cosmetics, textiles and hair dyes (Mori *et al.*, 1990).

The present work is a continuation of the preparation of new derivatives of anthra [1,2-*d*]imidazole-2,6,11–trione for biological application (Afrakssou *et al.*, 2011; Guimarães *et al.*, 2009). The reactivity of ethyl acetate bromide towards 1*H*-anthra [2, 1 - d] imidazole-2, 6, 11(3*H*)-trione under phase-transfer catalysis conditions using tetra *n*-butyl ammonium bromide (TBAB) as catalyst and potassium carbonate as base, leads to the formation of the title compound with good yields about 90% (Scheme 1).

The molecule of the title compound consists of three fused six-membered rings (A,B,*C*) and one five-membered ring (D) linked to two ethyl acetate groups as shown in Fig.1. The fused five and six-membered rings (C,D) are essentially planar with the largest deviation from the mean plane being -0.0185 (16) Å and built a dihedral angle of 3.07 (8) ° with (A) ring. The planes through the atoms forming each acetate group are nearly perpendicular to the mean plane of the anthra[1,2-*d*]imidazole system, as indicated by the dihedral angles between them of 79.94 (9) and 85.90 (9) °. The crystal packing displays intermolecular C—H···O no classic hydrogen bonding (Table 1).

### Experimental

To a solution of 1*H*-anthra [2,1-*d*]imidazole-2,6,11(3*H*)-trione (0.4 g, 1.51 mmol), potassium carbonate (1.56 g, 4.54 mmol) and tetra *n*-butyl ammonium bromide (0.62 g, 1.51 mmol) in DMF (20 ml) was added ethyl acetate bromide (0.41 ml, 3.78 mmol). Stirring was continued at room temperature for 24 h. The mixture was filtered and the solvent removed. The residue was extracted with water. The organic compound was chromatographed on a column of silica gel with ethyl acetate-hexane (1/1) as eluent. The crystals of the title compound were obtained by dissolving 50 mg of product in 5 ml of ethanol at about 363 K, followed by a slow evaporation of the solvent. The melting point of the isolated orange crystals is about 443 K.

### Refinement

All H atoms were located in a difference map and treated as riding with C—H = 0.95 Å for all aromatic H atoms, 0.99 Å for methylene and 0.98Å for methyl with *U*_iso_(H) = 1.2 *U*_eq_ aromatic and methylene and *U*_iso_(H) = 1.5 *U*_eq_ for methyl.

### Crystal data

**Table:**

C_23_H_20_N_2_O_7_	*F*(000) = 912
*M**_r_* = 436.41	*D*_x_ = 1.432 Mg m^−^^3^
Monoclinic, *P*2_1_/*c*	Melting point: 443 K
Hall symbol: -P 2ybc	Mo *K*α radiation, λ = 0.71073 Å
*a* = 17.462 (1) Å	Cell parameters from 5953 reflections
*b* = 13.0646 (9) Å	θ = 2.8–28.2°
*c* = 9.1411 (6) Å	µ = 0.11 mm^−^^1^
β = 103.915 (3)°	*T* = 100 K
*V* = 2024.2 (2) Å^3^	Needle, orange
*Z* = 4	0.49 × 0.11 × 0.09 mm

### Data collection

**Table:**

Bruker APEXII CCD diffractometer	5006 independent reflections
Radiation source: fine-focus sealed tube	3339 reflections with *I* > 2σ(*I*)
graphite	*R*_int_ = 0.057
φ and ω scans	θ_max_ = 28.3°, θ_min_ = 2.4°
Absorption correction: multi-scan (*SADABS*; Sheldrick, 2008*a*)	*h* = −23→17
*T*_min_ = 0.602, *T*_max_ = 0.746	*k* = −17→17
32055 measured reflections	*l* = −12→12

### Refinement

**Table:**

Refinement on *F*^2^	Primary atom site location: structure-invariant direct methods
Least-squares matrix: full	Secondary atom site location: difference Fourier map
*R*[*F*^2^ > 2σ(*F*^2^)] = 0.054	Hydrogen site location: inferred from neighbouring sites
*wR*(*F*^2^) = 0.153	H-atom parameters constrained
*S* = 1.01	*w* = 1/[σ^2^(*F*_o_^2^) + (0.0807*P*)^2^ + 0.7872*P*] where *P* = (*F*_o_^2^ + 2*F*_c_^2^)/3
5006 reflections	(Δ/σ)_max_ < 0.001
291 parameters	Δρ_max_ = 0.57 e Å^−^^3^
0 restraints	Δρ_min_ = −0.32 e Å^−^^3^

### Special details

**Table:**

Geometry. All e.s.d.'s (except the e.s.d. in the dihedral angle between two l.s. planes) are estimated using the full covariance matrix. The cell e.s.d.'s are taken into account individually in the estimation of e.s.d.'s in distances, angles and torsion angles; correlations between e.s.d.'s in cell parameters are only used when they are defined by crystal symmetry. An approximate (isotropic) treatment of cell e.s.d.'s is used for estimating e.s.d.'s involving l.s. planes.
Refinement. Refinement of *F*^2^ against all reflections. The weighted *R*-factor *wR* and goodness of fit *S* are based on *F*^2^, conventional *R*-factors *R* are based on *F*, with *F* set to zero for negative *F*^2^. The threshold expression of *F*^2^ > σ(*F*^2^) is used only for calculating *R*-factors(gt) *etc*. and is not relevant to the choice of reflections for refinement. *R*-factors based on *F*^2^ are statistically about twice as large as those based on *F*, and *R*- factors based on all data will be even larger.

### Fractional atomic coordinates and isotropic or equivalent isotropic displacement parameters (Å^2^)

**Table:**

	*x*	*y*	*z*	*U*_iso_*/*U*_eq_	
C1	0.03454 (11)	0.81216 (15)	0.1634 (2)	0.0212 (4)	
C2	−0.01666 (12)	0.88216 (16)	0.0739 (2)	0.0251 (4)	
H2	−0.0120	0.9531	0.0970	0.030*	
C3	−0.07428 (12)	0.84794 (18)	−0.0487 (2)	0.0291 (5)	
H3	−0.1089	0.8956	−0.1099	0.035*	
C4	−0.08148 (12)	0.74399 (17)	−0.0823 (2)	0.0300 (5)	
H4	−0.1204	0.7211	−0.1674	0.036*	
C5	−0.03235 (11)	0.67372 (16)	0.0073 (2)	0.0251 (4)	
H5	−0.0378	0.6027	−0.0153	0.030*	
C6	0.02552 (11)	0.70791 (15)	0.1315 (2)	0.0213 (4)	
C7	0.07787 (11)	0.63253 (15)	0.2280 (2)	0.0211 (4)	
C8	0.13726 (11)	0.67067 (15)	0.3619 (2)	0.0195 (4)	
C9	0.14819 (11)	0.77711 (14)	0.3921 (2)	0.0180 (4)	
C10	0.09975 (11)	0.85232 (15)	0.2866 (2)	0.0207 (4)	
C11	0.18249 (11)	0.59666 (14)	0.4533 (2)	0.0208 (4)	
H11	0.1729	0.5263	0.4296	0.025*	
C12	0.24099 (11)	0.62368 (15)	0.5779 (2)	0.0213 (4)	
H12	0.2722	0.5733	0.6399	0.026*	
C13	0.25240 (11)	0.72640 (15)	0.6087 (2)	0.0196 (4)	
C14	0.20748 (11)	0.80493 (14)	0.5205 (2)	0.0184 (4)	
C15	0.29890 (11)	0.87703 (15)	0.7166 (2)	0.0219 (4)	
C16	0.36449 (12)	0.71882 (15)	0.8400 (2)	0.0229 (4)	
H16A	0.3382	0.6635	0.8836	0.028*	
H16B	0.3880	0.7670	0.9221	0.028*	
C17	0.42943 (11)	0.67314 (16)	0.7757 (2)	0.0229 (4)	
C18	0.51981 (13)	0.53472 (18)	0.8019 (3)	0.0358 (5)	
H18A	0.5572	0.4993	0.8851	0.043*	
H18B	0.5506	0.5789	0.7491	0.043*	
C19	0.47416 (16)	0.4572 (2)	0.6938 (4)	0.0512 (7)	
H19A	0.4439	0.4135	0.7465	0.077*	
H19B	0.5109	0.4149	0.6539	0.077*	
H19C	0.4380	0.4926	0.6105	0.077*	
C20	0.21415 (12)	1.00388 (15)	0.5665 (2)	0.0238 (4)	
H20A	0.2225	1.0390	0.6651	0.029*	
H20B	0.1573	1.0081	0.5163	0.029*	
C21	0.26099 (12)	1.05796 (16)	0.4711 (2)	0.0250 (4)	
C22	0.29699 (17)	1.22304 (19)	0.4012 (3)	0.0454 (6)	
H22A	0.2759	1.2132	0.2915	0.054*	
H22B	0.3536	1.2046	0.4272	0.054*	
C23	0.2867 (2)	1.3309 (2)	0.4429 (3)	0.0564 (8)	
H23A	0.2304	1.3476	0.4199	0.085*	
H23B	0.3135	1.3761	0.3855	0.085*	
H23C	0.3096	1.3403	0.5510	0.085*	
N1	0.30651 (9)	0.77227 (12)	0.72586 (17)	0.0199 (3)	
N2	0.23663 (9)	0.89680 (12)	0.59127 (18)	0.0203 (3)	
O1	0.07231 (9)	0.54083 (10)	0.19817 (16)	0.0272 (3)	
O2	0.11351 (9)	0.94387 (11)	0.29568 (17)	0.0309 (4)	
O3	0.33699 (8)	0.94101 (11)	0.80032 (15)	0.0260 (3)	
O4	0.44572 (9)	0.70173 (12)	0.66182 (16)	0.0325 (4)	
O5	0.46530 (9)	0.59785 (12)	0.86323 (17)	0.0336 (4)	
O6	0.29952 (9)	1.01755 (12)	0.39486 (17)	0.0320 (4)	
O7	0.25385 (10)	1.15896 (12)	0.48532 (19)	0.0374 (4)	

### Atomic displacement parameters (Å^2^)

**Table:**

	*U*^11^	*U*^22^	*U*^33^	*U*^12^	*U*^13^	*U*^23^
C1	0.0234 (9)	0.0248 (10)	0.0172 (9)	−0.0005 (8)	0.0085 (7)	−0.0014 (7)
C2	0.0291 (10)	0.0255 (10)	0.0215 (10)	0.0010 (8)	0.0077 (8)	0.0019 (8)
C3	0.0274 (10)	0.0357 (12)	0.0234 (10)	0.0036 (9)	0.0044 (8)	0.0035 (9)
C4	0.0267 (10)	0.0387 (13)	0.0230 (10)	−0.0040 (9)	0.0030 (8)	−0.0016 (9)
C5	0.0251 (10)	0.0264 (11)	0.0252 (10)	−0.0045 (8)	0.0087 (8)	−0.0055 (8)
C6	0.0210 (9)	0.0261 (10)	0.0194 (9)	−0.0022 (8)	0.0097 (7)	−0.0007 (7)
C7	0.0241 (9)	0.0228 (10)	0.0193 (9)	−0.0035 (8)	0.0111 (8)	−0.0016 (7)
C8	0.0228 (9)	0.0199 (10)	0.0184 (9)	−0.0014 (7)	0.0099 (7)	−0.0012 (7)
C9	0.0203 (9)	0.0190 (9)	0.0167 (9)	0.0016 (7)	0.0084 (7)	0.0005 (7)
C10	0.0239 (9)	0.0214 (10)	0.0181 (9)	0.0005 (8)	0.0080 (7)	0.0001 (7)
C11	0.0278 (10)	0.0149 (9)	0.0216 (9)	−0.0014 (8)	0.0097 (8)	−0.0012 (7)
C12	0.0260 (10)	0.0210 (10)	0.0187 (9)	0.0026 (8)	0.0088 (8)	0.0033 (7)
C13	0.0207 (9)	0.0228 (10)	0.0170 (9)	0.0007 (7)	0.0078 (7)	0.0008 (7)
C14	0.0212 (9)	0.0168 (9)	0.0198 (9)	−0.0007 (7)	0.0099 (7)	−0.0004 (7)
C15	0.0229 (9)	0.0243 (10)	0.0207 (9)	0.0008 (8)	0.0096 (8)	0.0001 (8)
C16	0.0269 (10)	0.0257 (10)	0.0161 (9)	0.0015 (8)	0.0050 (8)	0.0018 (8)
C17	0.0216 (9)	0.0281 (11)	0.0178 (9)	0.0002 (8)	0.0023 (7)	0.0024 (8)
C18	0.0273 (11)	0.0401 (14)	0.0414 (13)	0.0138 (10)	0.0107 (10)	0.0111 (10)
C19	0.0367 (14)	0.0409 (16)	0.078 (2)	0.0058 (11)	0.0164 (14)	−0.0043 (14)
C20	0.0282 (10)	0.0188 (10)	0.0249 (10)	0.0003 (8)	0.0077 (8)	−0.0028 (8)
C21	0.0260 (10)	0.0241 (10)	0.0249 (10)	−0.0004 (8)	0.0063 (8)	−0.0003 (8)
C22	0.0604 (16)	0.0309 (13)	0.0533 (16)	−0.0024 (12)	0.0305 (13)	0.0111 (11)
C23	0.082 (2)	0.0395 (16)	0.0500 (17)	−0.0095 (15)	0.0199 (15)	0.0078 (13)
N1	0.0225 (8)	0.0196 (8)	0.0174 (8)	0.0010 (6)	0.0047 (6)	−0.0013 (6)
N2	0.0234 (8)	0.0179 (8)	0.0202 (8)	−0.0005 (6)	0.0062 (6)	−0.0014 (6)
O1	0.0325 (8)	0.0224 (8)	0.0270 (7)	−0.0027 (6)	0.0078 (6)	−0.0044 (6)
O2	0.0374 (8)	0.0211 (8)	0.0301 (8)	−0.0007 (6)	0.0000 (6)	0.0032 (6)
O3	0.0281 (7)	0.0247 (8)	0.0253 (7)	−0.0037 (6)	0.0067 (6)	−0.0054 (6)
O4	0.0330 (8)	0.0458 (10)	0.0213 (7)	0.0098 (7)	0.0116 (6)	0.0105 (6)
O5	0.0315 (8)	0.0438 (10)	0.0276 (8)	0.0139 (7)	0.0114 (6)	0.0144 (7)
O6	0.0389 (9)	0.0311 (8)	0.0304 (8)	−0.0011 (7)	0.0167 (7)	−0.0025 (6)
O7	0.0519 (10)	0.0224 (8)	0.0454 (10)	−0.0007 (7)	0.0261 (8)	0.0036 (7)

### Geometric parameters (Å, °)

**Table:**

C1—C6	1.394 (3)	C15—N2	1.401 (2)
C1—C2	1.397 (3)	C16—N1	1.447 (2)
C1—C10	1.492 (3)	C16—C17	1.519 (3)
C2—C3	1.388 (3)	C16—H16A	0.9900
C2—H2	0.9500	C16—H16B	0.9900
C3—C4	1.391 (3)	C17—O4	1.203 (2)
C3—H3	0.9500	C17—O5	1.326 (2)
C4—C5	1.383 (3)	C18—O5	1.469 (3)
C4—H4	0.9500	C18—C19	1.503 (4)
C5—C6	1.399 (3)	C18—H18A	0.9900
C5—H5	0.9500	C18—H18B	0.9900
C6—C7	1.482 (3)	C19—H19A	0.9800
C7—O1	1.227 (2)	C19—H19B	0.9800
C7—C8	1.487 (3)	C19—H19C	0.9800
C8—C11	1.393 (3)	C20—N2	1.456 (2)
C8—C9	1.422 (3)	C20—C21	1.508 (3)
C9—C14	1.413 (3)	C20—H20A	0.9900
C9—C10	1.489 (3)	C20—H20B	0.9900
C10—O2	1.219 (2)	C21—O6	1.201 (2)
C11—C12	1.380 (3)	C21—O7	1.335 (3)
C11—H11	0.9500	C22—O7	1.462 (3)
C12—C13	1.376 (3)	C22—C23	1.482 (4)
C12—H12	0.9500	C22—H22A	0.9900
C13—N1	1.383 (2)	C22—H22B	0.9900
C13—C14	1.419 (3)	C23—H23A	0.9800
C14—N2	1.400 (2)	C23—H23B	0.9800
C15—O3	1.217 (2)	C23—H23C	0.9800
C15—N1	1.376 (2)		
			
C6—C1—C2	119.54 (18)	N1—C16—H16B	109.3
C6—C1—C10	121.94 (17)	C17—C16—H16B	109.3
C2—C1—C10	118.47 (18)	H16A—C16—H16B	108.0
C3—C2—C1	119.95 (19)	O4—C17—O5	125.21 (19)
C3—C2—H2	120.0	O4—C17—C16	124.53 (18)
C1—C2—H2	120.0	O5—C17—C16	110.26 (16)
C2—C3—C4	120.1 (2)	O5—C18—C19	109.85 (19)
C2—C3—H3	119.9	O5—C18—H18A	109.7
C4—C3—H3	119.9	C19—C18—H18A	109.7
C5—C4—C3	120.47 (19)	O5—C18—H18B	109.7
C5—C4—H4	119.8	C19—C18—H18B	109.7
C3—C4—H4	119.8	H18A—C18—H18B	108.2
C4—C5—C6	119.52 (19)	C18—C19—H19A	109.5
C4—C5—H5	120.2	C18—C19—H19B	109.5
C6—C5—H5	120.2	H19A—C19—H19B	109.5
C1—C6—C5	120.32 (18)	C18—C19—H19C	109.5
C1—C6—C7	120.17 (17)	H19A—C19—H19C	109.5
C5—C6—C7	119.51 (18)	H19B—C19—H19C	109.5
O1—C7—C6	120.79 (17)	N2—C20—C21	112.12 (16)
O1—C7—C8	120.81 (18)	N2—C20—H20A	109.2
C6—C7—C8	118.40 (17)	C21—C20—H20A	109.2
C11—C8—C9	122.16 (17)	N2—C20—H20B	109.2
C11—C8—C7	116.35 (17)	C21—C20—H20B	109.2
C9—C8—C7	121.47 (17)	H20A—C20—H20B	107.9
C14—C9—C8	116.75 (16)	O6—C21—O7	124.74 (19)
C14—C9—C10	123.82 (17)	O6—C21—C20	125.98 (19)
C8—C9—C10	119.39 (16)	O7—C21—C20	109.28 (16)
O2—C10—C9	122.11 (17)	O7—C22—C23	107.4 (2)
O2—C10—C1	119.91 (17)	O7—C22—H22A	110.2
C9—C10—C1	117.94 (16)	C23—C22—H22A	110.2
C12—C11—C8	121.18 (18)	O7—C22—H22B	110.2
C12—C11—H11	119.4	C23—C22—H22B	110.2
C8—C11—H11	119.4	H22A—C22—H22B	108.5
C13—C12—C11	117.42 (17)	C22—C23—H23A	109.5
C13—C12—H12	121.3	C22—C23—H23B	109.5
C11—C12—H12	121.3	H23A—C23—H23B	109.5
C12—C13—N1	128.30 (17)	C22—C23—H23C	109.5
C12—C13—C14	123.76 (17)	H23A—C23—H23C	109.5
N1—C13—C14	107.94 (16)	H23B—C23—H23C	109.5
N2—C14—C9	135.76 (17)	C15—N1—C13	110.22 (15)
N2—C14—C13	105.53 (16)	C15—N1—C16	124.39 (16)
C9—C14—C13	118.71 (17)	C13—N1—C16	125.37 (16)
O3—C15—N1	127.91 (18)	C14—N2—C15	110.13 (15)
O3—C15—N2	125.93 (18)	C14—N2—C20	134.24 (16)
N1—C15—N2	106.15 (16)	C15—N2—C20	115.56 (16)
N1—C16—C17	111.62 (15)	C17—O5—C18	116.20 (16)
N1—C16—H16A	109.3	C21—O7—C22	116.27 (17)
C17—C16—H16A	109.3		
			
C6—C1—C2—C3	−2.2 (3)	C10—C9—C14—N2	3.2 (3)
C10—C1—C2—C3	175.18 (18)	C8—C9—C14—C13	0.8 (2)
C1—C2—C3—C4	0.4 (3)	C10—C9—C14—C13	−177.01 (17)
C2—C3—C4—C5	1.1 (3)	C12—C13—C14—N2	178.95 (17)
C3—C4—C5—C6	−0.7 (3)	N1—C13—C14—N2	−0.7 (2)
C2—C1—C6—C5	2.5 (3)	C12—C13—C14—C9	−0.9 (3)
C10—C1—C6—C5	−174.73 (17)	N1—C13—C14—C9	179.43 (16)
C2—C1—C6—C7	−178.23 (17)	N1—C16—C17—O4	21.5 (3)
C10—C1—C6—C7	4.5 (3)	N1—C16—C17—O5	−158.68 (16)
C4—C5—C6—C1	−1.1 (3)	N2—C20—C21—O6	15.8 (3)
C4—C5—C6—C7	179.67 (18)	N2—C20—C21—O7	−163.51 (17)
C1—C6—C7—O1	−177.49 (17)	O3—C15—N1—C13	−179.68 (19)
C5—C6—C7—O1	1.8 (3)	N2—C15—N1—C13	1.4 (2)
C1—C6—C7—C8	2.6 (3)	O3—C15—N1—C16	−1.2 (3)
C5—C6—C7—C8	−178.19 (17)	N2—C15—N1—C16	179.85 (16)
O1—C7—C8—C11	−3.0 (3)	C12—C13—N1—C15	179.94 (18)
C6—C7—C8—C11	176.95 (16)	C14—C13—N1—C15	−0.4 (2)
O1—C7—C8—C9	175.54 (17)	C12—C13—N1—C16	1.5 (3)
C6—C7—C8—C9	−4.5 (3)	C14—C13—N1—C16	−178.88 (16)
C11—C8—C9—C14	−0.1 (3)	C17—C16—N1—C15	−107.4 (2)
C7—C8—C9—C14	−178.58 (16)	C17—C16—N1—C13	70.9 (2)
C11—C8—C9—C10	177.81 (17)	C9—C14—N2—C15	−178.6 (2)
C7—C8—C9—C10	−0.6 (3)	C13—C14—N2—C15	1.6 (2)
C14—C9—C10—O2	7.6 (3)	C9—C14—N2—C20	4.7 (4)
C8—C9—C10—O2	−170.20 (18)	C13—C14—N2—C20	−175.14 (19)
C14—C9—C10—C1	−174.76 (16)	O3—C15—N2—C14	179.19 (18)
C8—C9—C10—C1	7.5 (3)	N1—C15—N2—C14	−1.8 (2)
C6—C1—C10—O2	168.12 (18)	O3—C15—N2—C20	−3.4 (3)
C2—C1—C10—O2	−9.2 (3)	N1—C15—N2—C20	175.55 (15)
C6—C1—C10—C9	−9.6 (3)	C21—C20—N2—C14	−95.1 (2)
C2—C1—C10—C9	173.13 (17)	C21—C20—N2—C15	88.3 (2)
C9—C8—C11—C12	−0.6 (3)	O4—C17—O5—C18	−10.5 (3)
C7—C8—C11—C12	177.95 (17)	C16—C17—O5—C18	169.61 (17)
C8—C11—C12—C13	0.5 (3)	C19—C18—O5—C17	−80.1 (2)
C11—C12—C13—N1	179.80 (18)	O6—C21—O7—C22	−0.2 (3)
C11—C12—C13—C14	0.2 (3)	C20—C21—O7—C22	179.08 (19)
C8—C9—C14—N2	−178.98 (19)	C23—C22—O7—C21	−175.2 (2)

### Hydrogen-bond geometry (Å, °)

**Table:**

*D*—H···*A*	*D*—H	H···*A*	*D*···*A*	*D*—H···*A*
C5—H5···O1^i^	0.95	2.49	3.353 (2)	151.
C12—H12···O6^ii^	0.95	2.56	3.380 (2)	145.
C16—H16A···O6^ii^	0.99	2.47	3.369 (3)	151.
C16—H16B···O4^ii^	0.99	2.22	3.120 (2)	151.

Symmetry codes: (i) −*x*, −*y*+1, −*z*; (ii) *x*, −*y*+3/2, *z*+1/2.
